# Effective Mechanical Properties of AlSi7Mg Additively Manufactured Cubic Lattice Structures

**DOI:** 10.1089/3dp.2021.0176

**Published:** 2022-08-03

**Authors:** Sara Mantovani, Mauro Giacalone, Andrea Merulla, Elena Bassoli, Silvio Defanti

**Affiliations:** ^1^ Department of Engineering “Enzo Ferrari,” University of Modena and Reggio Emilia, Modena, Italy.; ^2^ Ferrari S. p. A., Maranello, Italy.

**Keywords:** lattice structure, additive manufacturing, BCCxyz, compression test, energy absorption, finite element

## Abstract

Lattice structures, whose manufacturing has been enabled by additive technologies, are gaining growing popularity in all the fields where lightweighting is imperative. Since the complexity of the lattice geometries stretches the technological boundaries even of additive processes, the manufactured structures can be significantly different from the nominal ones, in terms of expected dimensions but also of defects. Therefore, the successful use of lattices needs the combined optimization of their design, structural modeling, build orientation, and setup. The article reports the results of quasi-static compression tests performed on BCCxyz lattices manufactured in a AlSi7Mg alloy using additive manufacturing. The results are compared with numerical simulations using two different approaches. The findings show the influence of the relative density on stiffness, strength, and on the energy absorption properties of the lattice. The correlation with the technological feasibility points out credible improvements in the choice of a unit cell with fewer manufacturing issues, lower density, and possibly equal mechanical properties.

## Introduction

Lattice structures are peculiar cellular solids obtained from the repetition of a unit cell in space. These structures have proven useful for heat transfer,^1^ thermal management,^2^ acoustic insulation,^3^ and mechanical load-bearing properties.^4,5^ For aerospace and automotive applications, the lattice structures are typically adopted as light core in sandwich structures^6^ or adopted to reinterpret the results of optimization analyses with a high target of stiffness-to-weight ratio.^7,8^ Furthermore, these structures are adopted in the biomechanical field to mimic the mechanical properties of bone tissue^9–11^ and to enhance bone regeneration.^12^

Due to the complexity of the lattices, traditional manufacturing methods are unsuitable for their production, whereas additive manufacturing (AM) fully deploys its capability to enable design freedom. Within the AM portfolio, laser-based powder bed fusion of metals (PBF-LB/M) is currently the most promising process for the integration of lattice structures in lightweight automotive components.^13,14^

Electron Beam Melting (EBM) represents an excellent alternative, with fewer geometrical difficulties,^15^ but it's very rarely applied to aluminum alloys because the peculiar thermal properties of this material make the EBM process extremely slow and complicated. PBF-LB/M allows the production of extremely complex hollow shapes with even lower costs than in the case of simple bulky geometries.^13^ In contrast, PBF-LB/M processes define new design constraints that in the case of trabecular structures are put to challenge.^16–19^

Three major limitations in this regard are as follows: the minimum downskin angle,^20–22^ the maximum overhang length, and the minimum manufacturable feature size.^13,23^ Since the removal of supports is unfeasible in the case of lattice structures, the periodic architecture should be designed and oriented onto the build platform so as to avoid areas whose downskin angle is lower than the critical overhang angle (COA).^22,24^ However, this choice would impose two hardly acceptable restrictions. The first would be the use of a limited set of unit cells that can be built with no overhangs, but woefully show highly anisotropic behavior, as for instance the BCCz cell.^25^

In addition, the avoidance of overhangs in a lattice is related to a specific orientation that might mismatch the manufacturing choices of the component the lattice is part of.^13^ Structures below the COA can still be built without supports only if the overhang length is within a certain limit.^20^ The maximum unsupported overhang length depends on the material, on the process parameters, and on the machine, since it is also affected by the mechanical interaction with the recoater. Piscopo *et al.*^26^ proved that parts made of AlSi10Mg alloy with a maximum overhang length of 6 mm, in combination with low curvature, can be produced with good quality, despite the downskin angle being lower than the COA.

Finally, lightweight lattice structures typically consist of thin struts or walls, which are needed to use small unit cells and pursue the maximum homogeneity of the component. In this regard, the design is limited by the minimum feature size that is several times higher than the width of the melt pool.^20^ As an example, robust walls can be produced with a thickness of two to three times the melt pool width. Calignano *et al.*^23^ discussed the manufacturability of thin walls in AlSi10Mg alloy by PBF-LB/M extensively. They found that, with optimized parameters, walls as thin as 0.2 mm can be accurately produced in the XY plane.

AM allows the designer to introduce lattice structures as functionally graded materials in the design of high-value structural components to be applied in aerospace^6^ and automotive fields.^27,28^ To do so, for a prescribed volume at a macroscopic scale, the average mechanical properties of the lattice structures are determined and referred to the properties of the bulk material. The mechanical properties of a lattice depend on the constituent material, on the unit topology, and on its relative density.^29,30^

The lattice structures are classified in strut-based cells^31–33^ and triply periodic minimal surface cells.^34–38^ In the literature, the experimental investigations and the numerical analyses are mainly performed on lattices with cubic cells. The topology of the periodic cell determines which dominant stresses affect the beams or the surfaces when the lattice is loaded. Hence, the strut-based lattices are beyond classified into *bending-dominated* and *stretch-dominated*^30^ using the Maxwell stability criterion.^39,40^ Stretch-dominated lattices exhibit higher specific compressive strength and modulus than the bending dominated structures; a detailed discussion is presented by Leary *et al.*^4^

For the reticular cells, a quantification of the average mechanical properties may be made theoretically,^41,42^ or numerically,^43–46^ or by experimental tests on manufactured samples.^4^

Several articles deal with the compressive failure modes of lattices. Quasi-static compressive test^47^ developed for porous and cellular metals is commonly adjusted to lattice materials. The compressive strength, the stiffness, and the specific energy absorption under compressive deformation are collected and compared as in Ref.^5^ Few contributions deal with tensile^48–52^ or bending^53^ tests.

Among the reticular lattices tested in the literature, the body-centered cubic (BCC) unit cell is most popular.^33,49,54–56^ Gümrük *et al.*^49^ test and compare a BCC lattice with a similar unit, with additional beams aligned with the direction of the tested compressive load (BCCz). Results of the compression tests show that the reinforced lattice (BCCz) has a stretch-dominated behavior, while the BCC works as a bending-dominated structure. Furthermore, the Young modulus of the BCCz lattice is 5 to 20 times higher than the Young modulus of the BCC. Similar results are presented in Leary *et al.*^33^

Wang *et al.*^55^ investigate the energy absorption of BCC and BCCz lattices. The two lattices are proposed in uniform density or in a graded density distribution. Results show that BCCz lattice absorbs roughly twice the energy absorbed by the BCC lattice, up to a deformation of 70%.

These results show that small interventions in the unit cell topology can substantially improve the mechanical performance of a lattice.^56^ In contrast, the mechanical properties of the BCCz are unbalanced toward the direction of the reinforcing struts, making these unit cells suitable for those applications in which the loading direction is well known. For a more general application, a lattice with quasi-isotropic stiffness and strength, a stable crushing behavior, and excellent energy absorption characteristics are welcome. Therefore, the present article focuses on BCC unit cell, reinforced with beams along the X, Y, and Z directions (BCCxyz), produced in AlSi7Mg using PBF-LB/M process. The same unit cell is also called in the literature as SC-BCC unit.^57,58^

The technological feasibility of the target structure was investigated by Sola *et al.*^13^: the lowest achievable relative density was 20% for a 3-mm cell size. Horizontal beams were characterized by dross and cracks, whereas inclined and vertical beams were produced accurately. The latter finding points out the importance of defects in determining the mechanical behavior of lattice structures produced by PBF-LB/M, which is consistent for instance with the results by Yan *et al.*^34^ who observe cracks affecting the behavior of gyroid lattices.

The construction of thin struts and walls, sometimes beyond the self-supporting limitations, may result as feasible but cause defects that make the effective geometry significantly different from the nominal one. Melt pool instability, shrinkage, and the dross effect^21^ may cause, at worst, the job crash; otherwise, they can lead to cracks, uneven feature size, and massive presence of satellite particles.^13^ It is therefore mandatory to assess the manufacturing boundaries of the lattice structures, both in terms of feasibility and of deviation from the nominal geometry and expected behavior. The topic is thoroughly addressed by several authors^59–62^ through an approach that relies on micro-tomography for the construction of nonideal finite element (FE) models.

Although extremely accurate, this methodology might be inapplicable in industrial environments for big components. In these cases, the identification of correction coefficients to be applied to ideal models might be a more effective solution. The quick increase of the computational effort with the increase in the size of the lattice structure is well spotted by Smith *et al*.^63^

In this article, the same lattice structures as in Sola *et al.*^13^ are tested under quasi-static compression to assess the multifunctional capability of these structures for energy absorption and load bearing applications, for varying relative density. The experimental results are compared to the analytical and the FE forecasts.

## Methods

### Unit cell topology

Maxwell's stability criterion may be used to determine the nature of the loads affecting the lattice beams. The criterion is applied to a frame, which is similar to the original lattice, but has pin joints at the connections of the beams, instead of the rigid joints in the lattice.

So, for any three-dimensional frame composed by *s* beams joined at *n* nodes the value of Maxwell number *M* is evaluated as:

(1) M=s−3n+6.

If M<0, the frame is not statically determinate and acts like a mechanism under an external load. The corresponding lattice is held up together by the transmission of bending moments at the joints between the beams: it is thus defined as a bending-dominated lattice.

If M=0, the frame has the minimum number of beams to be statically determinate; all the beams in the frame absorb any external load with axial stresses. The beams in the corresponding lattice may be loaded with some bending moments, but the entity of such moments is substantially lower compared with the axial forces. Therefore, the lattices may be defined as stretch dominated.

If M>0, the frame has more beams than those required to be statically determinate. Like the lattices with M=0, these lattices exhibit stretch-dominated behavior, with higher elastic properties given by the superior number of beams.

Ashby^30^ showed that the relative stiffness ($E∕E_{S}$) and relative strength ($\sigma_{y}∕\sigma_{ys}$) of stretch-dominated lattices are proportional to the lattice relative density ($\rho ∕\rho_{s}$) and are typically higher than their bending dominated counterparts.

Mazur *et al.*^25^ compare the mechanical properties of cubic lattice cells manufactured by SLM in TI6Al4V and AlSi12Mg. FCCz structures show the highest specific elastic modulus. Conversely, the BCC structures show the lowest modulus, because of their under-stiff behavior (*M* < 0), which leads to high structural compliance.

**FIG. 1.**
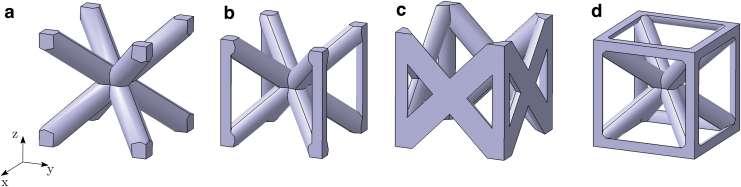
Library of truss-based unit cells ($\rho ∕\rho_{s}$ = 0.2): **(a)** BCC, **(b)** BCCz, **(c)** FCCz, **(d)** BCCxyz. BCC, body-centered cubic. Color images are available online.

Wang *et al.*^64^ compare three cell configurations in 316L, namely BCC, BCCz, and FCCz (Fig. 1a–c). All structures demonstrated a stretch-dominated deformation mode and experienced stable plastic deformation before densification. Deformation of the structures with Z-struts progressed by buckling of vertical struts with development of plastic hinges in regions near nodes, whereas the deformation of Z-strut-free lattice structures proceeded only through the development of plastic hinges near nodes and the rotation of slanted struts around the nodes.

The lattice under examination in this article has a BCCxyz unit cell (Fig. 1d). Its corresponding frame has 20 beams and 9 joints, leading to a Maxwell Number M=−1. The frame has thus a pliability, which is described by Lake and Klang^41^ as: “the repeating cell […] is not a kinematically stable truss. It has no torsional stiffness because all diagonals intersect at one point.” However, two adjacent unit cells have 36 beams and 14 nodes, which lead to M=0. Therefore, a domain full of BCCxyz cells has a stretch-dominated behavior.

### Relative density

The relative density ($\rho ∕\rho_{s}$) expresses the ratio between the density of the lattice and the density of the constituent material. Relative density has a major influence on the mechanical properties of the lattice.

Given the unit cell topology, the relative density depends solely on the strut diameter (*d*) and the unit cell size (*l*). Gibson and Ashby^29^ calculate the relative density by considering the beams as perfect cylinders.

Applying this analytical method to the BCCxyz unit cell, one obtains:

(2) $${\left[\frac{\rho }{\rho_{s}}\right]}_{Ashby}=\frac{\pi }{4}\left(3+4\sqrt{3}\right){\left(\frac{d}{l}\right)}^{2}.$$

This model overestimates the actual relative density of the lattice, as the volumes of the perfect beams overlap at the joining nodes.

In this study, the nominal relative density was computed using commercial CAD software at 22 nominal values of d∕l between 0 and 5/6. The resulting values are named hereafter as $\rho_{CAD}$. The density of the bulk material $\rho_{s}$was assumed to be 2.67 × 10^3^ kg/m^3^. The ratio $\rho_{CAD}∕\rho_{s}$ is fit by the polynomial function:

(3) $$\begin{array}{c} \frac{\rho_{CAD}}{\rho_{s}}=8.432{\left(\frac{d}{l}\right)}^{4}-17.960{\left(\frac{d}{l}\right)}^{3}\\ +10.900{\left(\frac{d}{l}\right)}^{2}-0.295\left(\frac{d}{l}\right). \end{array}$$

**FIG. 2.**
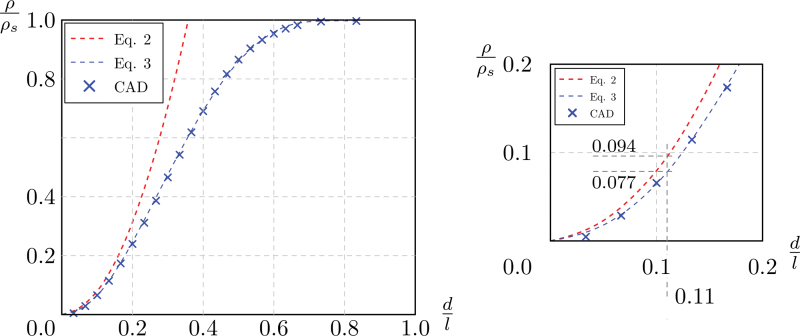
Relative density of BCCxyz lattice. Color images are available online.

Figure 2 shows $\rho ∕\rho_{s}$ computed with Equations (2) and (3), the error between the resulting relative densities is higher than 20%, at $\rho ∕\rho_{s}$ above 0.11.

Table 1 compares, in the third and fourth column, the two values of relative density of the specimens tested in this study.

**Table 1.** Results for Density, Stiffness, Strength, and Energy Absorption

Sample	d* [*mm]	$\rho ∕\rho_{s}$			E* [*MPa]			$10^{3}\left(\frac{E}{E_{s}}\right)\left(\frac{\rho }{\rho_{s}}\right)$CB	σ_r_* [*MPa]	$10\left(\frac{\sigma_{r}}{\sigma_{ys}}\right)\left(\frac{\rho }{\rho_{s}}\right)$	$\varepsilon_{dens}$	W* [*MJ/m^3^]	W_eff_* [*%]
		Equation (2) [29]	CAD	exp [13]	FE	DIC	CB						
3.2.1	0.5	0.2	0.177	0.221	2330	1020	1090	7.04	13.2	4.61	0.60	5.76	65.3
3.2.2						930	890	5.68	13.4	4.62	0.54	6.01	65.7
3.2.3						980	990	6.43	13.9	4.88	0.60	6.15	69.8
3.2.4						990	750	4.84	15.1	5.24	0.60	6.77	72.3
Average						**980**	**930**	**6.00**	**13.9**	**4.84**	**0.59**	**6.17**	**68.2**
St. dev.						37	145	0.95	0.8	0.30	0.03	0.43	3.4
3.3.1	0.6	0.3	0.243	0.267	3010	1970	1940	101.00	19.7	5.53	0.57	10.09	61.5
3.3.2						1992	1040	56.22	19.0	5.52	0.47	9.73	70.4
3.3.3						942	1100	61.22	16.5	4.94	0.58	8.17	71.6
3.3.4						2030	1730	90.20	17.8	5.00	0.56	9.59	68.7
Average						**1734**	**1453**	**77.16**	**18.2**	**5.25**	**0.55**	**9.39**	**68.0**
Standard deviation						528	451	21.84	1.4	0.32	0.05	0.84	4.5
3.4.1	0.7	0.4	0.315	0.318	4150	2840	2250	101.10	29.0	7.02	0.55	14.89	68.2
3.4.2						2660	2470	110.00	28.1	6.74	0.45	16.05	61.8
3.4.3						2690	2680	121.90	29.4	7.20	0.57	14.79	68.6
3.4.4						1740	1490	66.70	27.9	6.72	0.55	14.27	70.1
Average						**2483**	**2223**	**99.93**	**28.6**	**6.92**	**0.53**	**15.00**	**67.1**
Standard deviation						501	519	23.73	0.8	0.23	0.05	0.75	3.7

CB, crossbar; DIC, Digital Image Correlation; FE, Finite Element.

### Specimens and technological feasibility

The BCCxyz unit cell is 3 mm in size, with increasing nominal diameter of the beam (from 0.5 to 0.7 mm) for increasing relative density. The specimens are cubes containing 16-U cells per side, named as in Table 1 in Stiffness and Strength section. The specimens are built in an AlSI7Mg alloy by PBF-LB/M, in an SLM 500 machine (SLM Solutions Group AG, Lübeck, Germany) operated with a scan speed of 1100 mm/s, laser power of 350 W, and layer thickness of 0.050 mm. After printing, all the specimens were T6 treated under an inert atmosphere, which implied heating at 540°C for 16 h, water quenching down to 30°C/35°C, and artificial aging at 160°C for 10 h.

The manufacturing procedure was the same as in Sola *et al.*,^13^ where the lattices with beam diameter lower than 0.5 mm, even if theoretically compliant with technical specifications of the AM machine, resulted in unstable structures. They are therefore excluded from the present tests.

Sola *et al.*,^13^ propose the nominal density of the specimens as by Gibson and Ashby and calculated as in Equation (2). Effective density of all the specimens was also assessed experimentally, and the results are recalled here in the fifth column of Table 1. In view of the more accurate calculation of the nominal density $\rho_{CAD}$, the considerations in Sola *et al.*^13^ need to be revised. The actual density of the specimens is very close to the nominal one, especially for the specimens 3.4 and 3.3. The lighter specimens (3.2) are built with a 25% extra weight with respect to the CAD geometry.

Even with the new calculation proposed here, the upper lightweighting limit for a cell dimension of 3 mm remains substantially unchanged at 80%. Within the feasibility window, horizontal struts are found affected by frequent vertical cracks that propagate from the downskin surfaces, which pose doubts on their effective contribution to the mechanical response and failure modes.

**FIG. 3.**
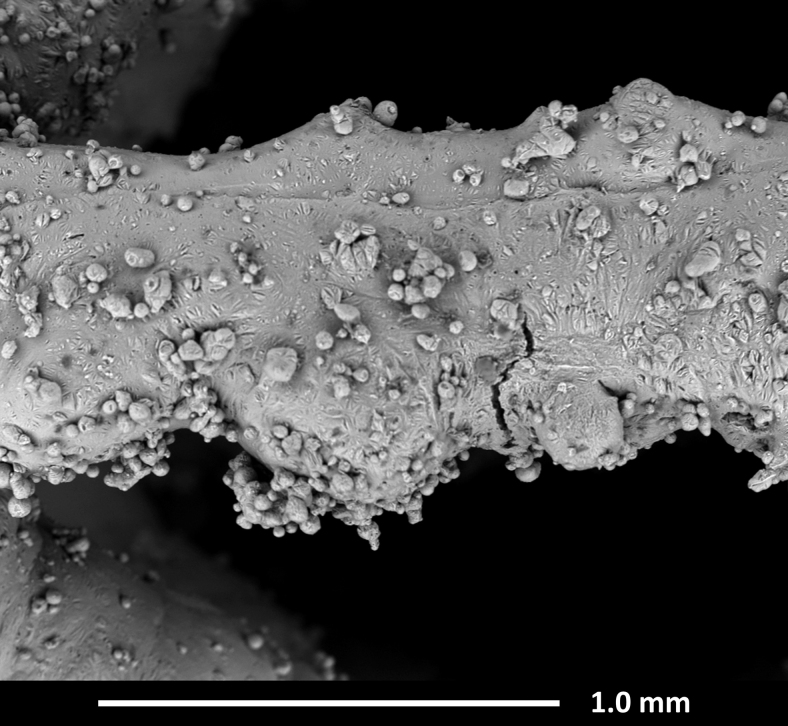
SEM image showing dross and cracks in a horizontal beam of a 3.2 specimen. SEM, scanning electron microscope.

At the same time, the dross defect causes, if compared to the nominal geometry, the presence of extra material that might not contribute fully to the mechanical performance of the structure. An example of the defects in horizontal beams is shown in Figure 3, acquired using a scanning electron microscope (Quanta-200; FEI, The Netherlands) to observe a 3.2 specimen from the side. The build direction Z is vertical in the image.

### Numerical modeling

Any uniform lattice structure may be condensed in a uniform elastic continuum. In the linear elastic field, the stresses are linked to the strains by the linear tensor $\underline{C}$:

(3a) $$\left\{\begin{array}{c} \sigma_{x}\\ \sigma_{y}\\ \sigma_{z}\\ \tau_{xy}\\ \tau_{yz}\\ \tau_{zx} \end{array}\right\}=\left[\begin{array}{cccccc} c_{11} & c_{12} & c_{13} & c_{14} & c_{15} & c_{16}\\ c_{21} & c_{22} & c_{23} & c_{24} & c_{25} & c_{26}\\ c_{31} & c_{32} & c_{33} & c_{34} & c_{35} & c_{36}\\ c_{41} & c_{42} & c_{43} & c_{44} & c_{45} & c_{46}\\ c_{51} & c_{52} & c_{53} & c_{54} & c_{55} & c_{56}\\ c_{61} & c_{62} & c_{63} & c_{64} & c_{65} & c_{66} \end{array}\right]\left\{\begin{array}{c} \varepsilon_{x}\\ \varepsilon_{y}^{}\\ \varepsilon_{z}\\ \gamma_{xy}\\ \gamma_{yz}\\ \gamma_{zx} \end{array}\right\}$$

(3b) $$\underline{\sigma }=\underline{C}\underline{\varepsilon }$$

Lake and Klang^41^ show the influence of rotational symmetries on the mechanical properties of a generic lattice structure. The rotational symmetries in the BCCxyz lattice lead to the conclusion that it globally behaves as an orthotropic material. As well as that, only three elements in the constitutive tensor *C* are independent. These elements may be expressed in terms of three elastic constants, namely Young's Modulus (*E*), Poisson's Ratio (*ν*), and shear modulus (*G*).

In this study, the three main elastic constants of the BCCxyz lattice are obtained as functions of the relative density, adopting a numerical homogenization technique.^43,45^ The commercial solver MSC Marc 2017 is used in all simulations, under the assumption of small strains and linearly elastic material. Thus, nine FE models are created with the unit cell size of 5 mm and an increasing diameter for the beams. The unit cell is modeled adopting first-order tetrahedral elements. Periodic boundary conditions are then introduced on the FE models to simulate a single unit cell in an infinite lattice domain; these kinematic conditions are presented in Eqns. (17) and (25) of Sun and Vaidya.^43^

Besides the FE models adopting solid elements, the same unit cells are also modeled by Timoshenko beam elements. The average element size for both modeling techniques is 0.2 mm. Each FE model with solid elements consists of about 60,000 nodes and 300,000 elements, while each beam-like model consists of about 600 nodes and 600 elements.

### Experimental testing

**FIG. 4.**
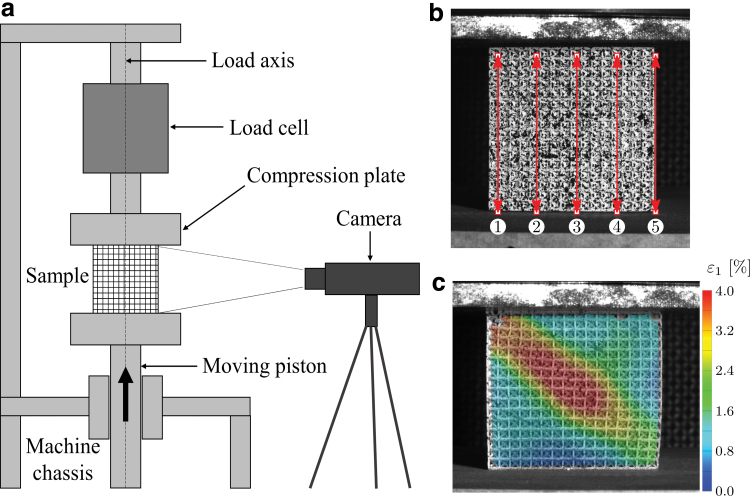
**(a)** Sketch of the experimental setup, **(b)** virtual extensometers used in DIC measurements, **(c)** maximum principal strain contour of specimen 3.3.3 at compression ratio of 6%. DIC, digital image correlation. Color images are available online.

ISO 13314-2011^47^ is the reference standard adopted during the experimental campaign. The compression tests are performed using a SCHENCK HYDROPULS PSB universal testing machine, mounting a 250 kN load cell; the experimental setup is sketched in Figure 4a. Each test is carried with a constant crosshead velocity of 3 mm/min leading to an initial strain rate of 10^−3^ s^−1^.

Herein, Digital Image Correlation (DIC) technique is used to measure the engineering strain at the early phases of the tests, in the linear deformation region at the beginning of the compressive stress–strain curve.^47^ The DIC technique adopts high resolution cameras to film the entire test. The face of the sample is painted in white and speckled with black acrylic paint to provide sufficient information to the digital images.

The images are then postprocessed to evaluate the local strain on the lattice beams.^52,65,66^ In this study, the DIC system uses a PointGrey^®^ Grasshopper3 camera with a SONY^®^ IMX174 sensor, with an acquisition frequency of 10 Hz. Ten points on the front face of the sample are identified to define five virtual extensometers aligned with the loading direction (Fig. 4b). Then, the deformation on the virtual extensometers is measured. Finally, the resulting quasi-elastic gradients are averaged for comparison with the results obtained using the displacement of the crossbar (CB) for the calculation of strain. GOM Correlate 2019 DIC software is used for the postprocessing. The maximum principal strain contour map is shown in Figure 4c.

The following quantities are calculated by processing the experimental data as prescribed by ISO 13314-2011^47^:

○ Quasi-Elastic Gradient (*E*): gradient of the straight line determined within the linear deformation region at the beginning of the compressive stress–strain curve.
○ First Maximum Compressive Strength $\left(\sigma_{r}\right)$: compressive stress corresponding to the first local maximum in the stress–strain curve.
○ Plateau Stress $\left(\sigma_{pl}\right)$: arithmetical mean of the stresses at 0,1% strain intervals between or 25% and 40% compressive strain. The starting point of the range, suggested at 20% in the standard, is raised to 25% in this study, to exclude from the calculation the instability that these samples show for deformations up to 20%.
○ Engineering strain at the onset of densification ($\varepsilon_{dens}$).
○ Volumetric Energy Absorption up to a strain of 50% (*W*): area under the stress–strain curve up to 50% strain.
○ Crash Force Efficiency up to a strain of 50% ($W_{eff}$): *W* divided by the product of the maximum compressive stress within the strain range and the magnitude of the strain range.

Furthermore, the following quantities are computed:

○ Normalized Specific Stiffness ($\frac{E}{E_{s}}\frac{\rho_{s}}{\rho }$)
○ Normalized Specific Compressive strength ($\frac{\sigma_{r}}{\sigma_{ys}}\frac{\rho_{s}}{\rho }$)

*E_s_*, $\sigma_{ys}$, and $\nu_{s}$ are the Young Modulus, yield strength, and Poisson's ratio of the reference AlSi7Mg alloy, which were assumed as 70 GPa, 130 MPa, and 0.33, respectively.

## Results and Discussion

### Numerical simulations

**FIG. 5.**
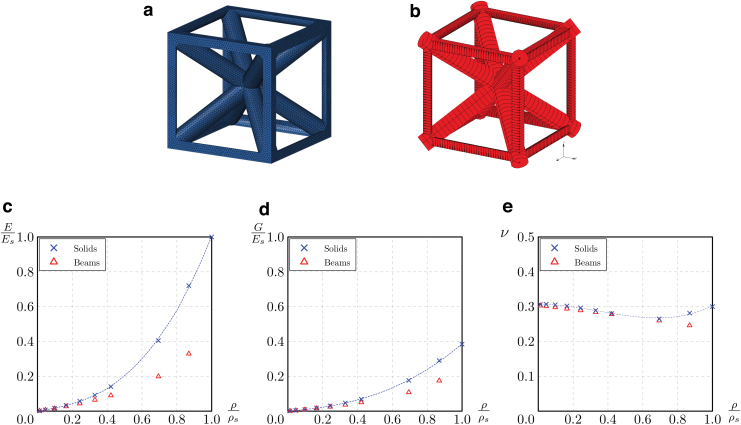
FE models of the unit cells with solid elements **(a)** and beam elements **(b)**, relative Young's modulus **(c)**, shear modulus **(d)**, and Poisson's ratio **(e)** of BCCxyz lattice as computed by numerical simulations. FE, finite element. Color images are available online.

Figure 5 compares the results of the FE simulations performed with first-order tetrahedral elements (Fig. 5a) and Timoshenko beam elements (Fig. 5b). At $\rho ∕\rho_{s}$ below 0.1, good correspondence is found between the two FE models, with an error lower than 10%. For $\rho ∕\rho_{s}$ above 0.1, the slenderness ratio of the beams decreases, and beam theory becomes inadequate for the lattice struts. This leads to a substantial difference between the results obtained from the models with beam elements and those with solid elements.

The modeling technique using solid elements is more reliable and is therefore adopted hereafter as the reference for the comparison with experimental data.

The numerical results consider the relative density ranging from 0 to 1, under the following two assumptions^45^:

- If $\rho ∕\rho_{s}=0$ (i.e., the volume of the lattice is completely void), the mechanical properties are zero.
- If $\rho ∕\rho_{s}=1$(i.e., the volume of the lattice is completely full), the mechanical properties of the lattice coincide with those of the bulk material.

Thus, the results of the numerical homogenization performed with these elements are approximated with the following functions:

(4) $$\frac{E}{E_{s}}=\frac{41}{60}{\left(\frac{\rho }{\rho_{s}}\right)}^{3}+\frac{17}{93}{\left(\frac{\rho }{\rho_{s}}\right)}^{2}+\frac{13}{94}\left(\frac{\rho }{\rho_{s}}\right).$$

(5) $$\frac{G}{E_{s}}=\left[\frac{1}{2\left(1+\nu_{s}\right)}\right]\left[\frac{5}{13}{\left(\frac{\rho }{\rho_{s}}\right)}^{3}+\frac{4}{9}{\left(\frac{\rho }{\rho_{s}}\right)}^{2}+\frac{17}{97}\left(\frac{\rho }{\rho_{s}}\right)\right].$$

(6) $$\nu =\frac{23}{88}{\left(\frac{\rho }{\rho_{s}}\right)}^{3}-\frac{3}{11}{\left(\frac{\rho }{\rho_{s}}\right)}^{2}+\frac{1}{154}\left(\frac{\rho }{\rho_{s}}\right)+\frac{31}{101}.$$

### Mechanical tests

#### Stiffness and strength

**FIG. 6.**
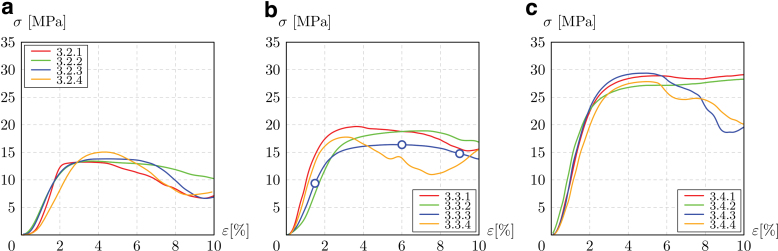
Compressive stress–strain diagrams, up to the first maximum compressive strength of 3.2 **(a)**, 3.3 **(b)**, and 3.4 **(c)** samples. Color images are available online.

Figure 6 shows the elastic region and the initial field of irreversible deformation in the curves, up to the $\sigma_{r}$. The strain plotted in the graphs is that obtained from the CB displacement. Stiffness, namely quasi-elastic gradient, and strength are calculated as in the ISO 13314-2011^47^ and are listed in Table 1 for all the samples.

In all the three density groups, a significant statistical dispersion is found in the quasi-elastic gradient of the lattice, with the standard deviation reaching 30% of the average value. This scattering may be reduced by introducing a preliminary test phase in which the sample is loaded and then unloaded, as performed by Mazur *et al.*^25,67^

The quasi-elastic gradient measured by DIC brings contrasting results. The average values calculated by DIC are generally higher than using the CB displacement, with a deviation between the two results that is not excessive. Yet, the high scattering of DIC suggests a less robust measurement, likely due to the fact that DIC detects the strain field on the frontal face of the samples. The deformation on this face may be different from that within the sample, since the top and bottom of the sample are not perfectly planar.

Results on the strength of the lattice have little statistical dispersion and show a general increase in strength along with the relative density, as envisaged by the theory.

**FIG. 7.**
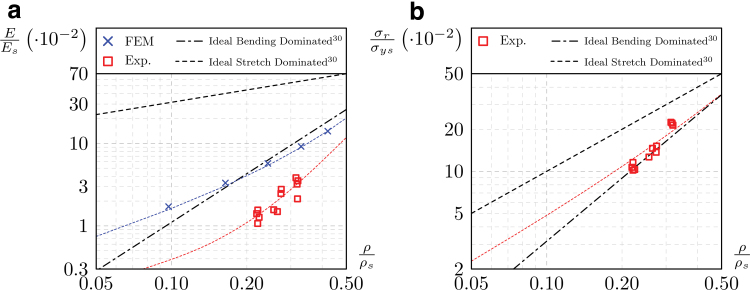
General tendency of relative stiffness **(a)** and strength **(b)**. Color images are available online.

The global trend of relative stiffness is shown in Figure 7a, where the experimental quasi-elastic gradients (CB) are compared with the theoretical curves proposed by Ashby^30^ and with the results of the numerical simulations discussed in Numerical Simulations section. The numerical simulations overestimate the experimental results by 60–100%. This is likely explained by the horizontal beams being partially compromised by defects, as shown in Figure 3. More generally, the result quantifies the deviation between the mechanical response of the manufactured parts and that of the nominal geometries, thus providing reliable data for the adoption of correction factors in the design of lattice components.

Previous studies^51,59,62^ come to similar results and evidence the shortage of ideal FE models in describing a failure behavior that is highly affected by stress concentrations. Nevertheless, purpose of the present contribution is to identify a straightforward approach for the prevision of the mechanical response, in all the cases where a microtomography and nonideal FE modeling might not be compatible with industrial needs.

In previous studies on Ti6Al4V lattices fabricated by LB-PBF/M,^54^ the experimental data, the analytical prediction, and the FE results were found in better agreement, presumably because of bigger cell and beam dimensions, which are less critical for the additive process than those considered here.

The resistance of the lattice samples lies between the curves defining the ideal stretch-dominated and bending-dominated behavior (Fig. 7b). Even though the lattice under examination is considered as stretch dominated, the overall tendency of the experimental results is coherent with the bending-dominated behavior. This is also due to the fact that beam theory is not applicable for the lattice in this study.

In the literature,^32^ the compressive response of several lattice structures produced in TiAl6V4 is correlated to the increasing relative density using a power law. Herein, under the same assumptions made for Equations (4)–(6), the relative stiffness and relative strength of the lattice can be described as a function of the relative density by the following polynomials:

(7a) $$\frac{E}{E_{s}}=\frac{31}{27}{\left(\frac{\rho }{\rho_{s}}\right)}^{3}-\frac{7}{36}{\left(\frac{\rho }{\rho_{s}}\right)}^{2}+\frac{2}{43}\left(\frac{\rho }{\rho_{s}}\right).$$

(7b) $$\frac{\sigma_{r}}{\sigma_{ys}}-\frac{43}{75}{\left(\frac{\rho }{\rho_{s}}\right)}^{2}+\frac{32}{75}\left(\frac{\rho }{\rho_{s}}\right).$$

The coefficient of determination (*R*^2^) for the two equations is of 0.9997 and 0.9931, respectively.

#### Failure mechanism

**FIG. 8.**
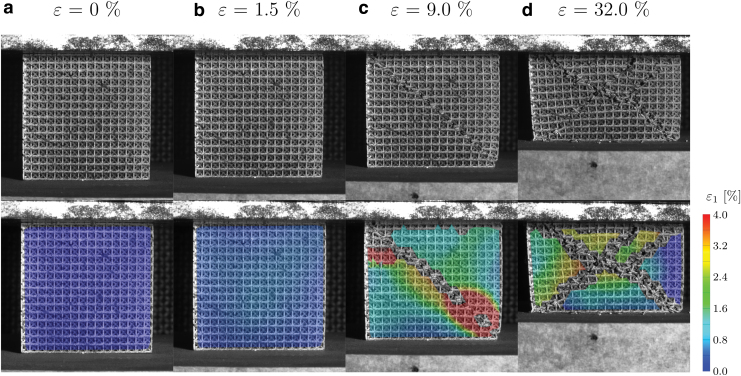
Failure progression and maximum principal strain contour of specimen 3.3.3 at the following compression ratios: **(a)** 0%, **(b)** 1.5%, **(c)** 9.0%, and **(d)** 32.0%. Color images are available online.

**FIG. 9.**
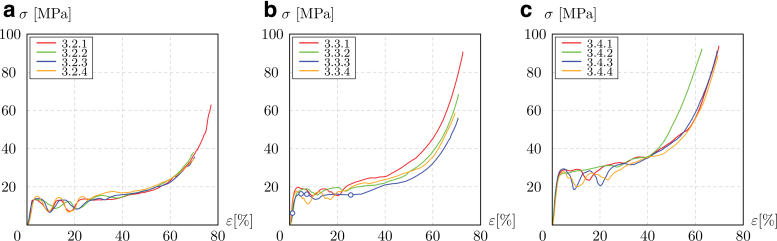
Complete compressive stress–strain response of 3.2 **(a)**, 3.3 **(b)**, and 3.4 **(c)** samples. Color images are available online.

DIC allows the synchronized analysis of the failure phenomena and of the stress–strain curves. Figures 8 and 4c show the main failure steps of sample 3.3.3, occurring at compressive ratios of 1.5%, 6.0%, 9.0%, and 32%. These steps are highlighted in the stress–strain curve, Figure 9b.

All the samples experience the same macroscopic failure mechanism. After the quasi-elastic region (Fig. 8b), the first failure is detected at the horizontal beams, in which the effect of the tensile loads is magnified by the presence of several cracks compromising the beam sections.

At a macroscopic level, all the tested samples exhibit a slide along a plane inclined by 45° with respect to the load direction (Fig. 8c) similar to what was observed by Gavazzoni *et al.*^58^ This slide is directly linked to the first fall in the compressive stress of the lattice, which usually occurs for strains between 5% and 15% (Fig. 9).

After the first slide is completed, the load starts rising again as compression proceeds, until a second slide is activated along a second plane, inclined by −45° with respect to the load direction (Fig. 8d), along with a second fall in the stress values.

At the end of the second slide, the specimen is divided in 4 wedge-shaped parts, which are crushed together while the compressive deformation advances, and as the test continues the sample starts the densification phase.

The deformation progression differs from the findings by Wang *et al.*,^64^ who observe a progressive collapse without any shear band of BCCz and FCCz lattices manufactured in 316L. This is most likely due to the diameter of the vertical beams. Adopting a lattice with higher relative density, and thus higher beam diameters, may result in a more progressive collapse.

#### Energy absorption

Figure 9a–c shows the full response of the lattice, from the area of elastic behavior to that of permanent deformation. The curves present the typical behavior of plastic foams; the linear elastic region smoothly turns into the plateau, which is then followed by a fast increase once the densification occurs. At the beginning of the plateau region, almost all the samples exhibit a reversed camel-back response due to the double diagonal slide occurring during the compression.

In the region of irreversible strain, the stress remains nearly constant for samples 3.2 and 3.3, while the denser samples always exhibit a slight increase in stress. Finally, the plateau region smoothly ends toward the densification phase. The determination for the densification strain ($\varepsilon_{dens}$) is not simple, since there is no neat passage from plateau to densification.

Table 1 collects the results on $\sigma_{pl},\varepsilon_{dens}$, and *W*. As $\rho ∕\rho_{s}$ increases, the plateau stress and the total energy absorption increase. The crash force efficiency ($W_{eff}$), on the other hand, remains almost constant.

## Conclusions

In this work, the compressive elasto-plastic response of a uniform aluminum lattice structure produced by PBF-LB/M is presented. The experimental elastic properties of the structures are compared to the FE simulations, which are performed with two alternative approaches, the first adopting three-dimensional elements and the second adopting Timoshenko beam elements.

The feasibility study shows that the beams aligned with the building direction are successfully constructed; the diagonal beams are built with some minor downskin effects, while the nonself-supporting horizontal beams present severe cracks, extensive dross, and uneven diameter. These cracks lead to early failure of the horizontal beams, which experience a tensile load when the lattice is loaded in compression along a vertical direction. Therefore, the structural contribution of the horizontal struts is limited, if compared to the counterpart of the other beams.

During the collapse, the lattice exhibits a double diagonal slide, which influences the stress–strain response at strains below 0.25.

The increase of the relative density in the lattice brings about an increase in stiffness, strength, and plateau stress. An increase in energy absorption and specific energy absorption properties is also encountered alongside the increase in relative density.

The energy absorption properties of the BCCxyz lattice are still promising in designing mechanical components deputed to absorb energy through plastic deformation.

Some interesting points emerge from the experimental results.

The structural contribution of the defective beams along directions x and y is still uncertain. The mechanical properties of a BCCz unit cell could be evaluated in future research works. Furthermore, the interaction between the lattice and solid material in practical applications should be investigated and mimicked by a further detailed FE and experimental campaign.
